# Caregivers’ use of insecticide-treated nets is associated with the use of ITNs by children under the age of five in Ghana

**DOI:** 10.1371/journal.pone.0280065

**Published:** 2023-01-06

**Authors:** Daudi Yeboah, Michael Boah, Martin Nyaaba Adokiya

**Affiliations:** Department of Epidemiology, Biostatistics and Disease Control, School of Public Health, University for Development Studies, Tamale, Ghana; Fundação Oswaldo Cruz Centro de Pesquisas René Rachou: Fundacao Oswaldo Cruz Instituto Rene Rachou, BRAZIL

## Abstract

**Background:**

Malaria poses a greater risk to children under the age of five years due to its high morbidity and mortality rates. The use of Insecticide-Treated Net (ITN) has been proven to be an effective preventive intervention in the control of malaria. However, its utilisation remains low. This study assessed the association of mother or caregiver’s utilisation of ITN on its use by their children under five years of age in Ghana.

**Methods:**

This study used data from the 2019 Ghana Malaria Indicator Survey (GMIS). The study analysed a weighted sample of 1,876 women aged 15–49 years who had at least one child under the age of five. In this study, the outcome variable is mosquito bed net use in children under five years. We performed descriptive statistics, chi-square tests, and multinomial logistic regressions.

**Results:**

Of the women studied, 58.59% [95% CI: 55.39, 61.71] slept under mosquito bed nets the previous night. The utilisation of ITN in children under five was 61.88% [95% CI: 58.43, 65.2] on the night before the study. The adjusted logistic regression revealed that mothers/caregivers who slept under a mosquito bed net were more likely to have their children under five years of age sleeping under a mosquito bed net (RRR = 2.47, 95% CI: 1.48, 4.12; p <0.001). In addition, the use of ITN in children under five was also found to be predicted by the number of ANC visits, the number of children under five in the household, and wealth status.

**Conclusion:**

The study found that the use of ITN by mothers/caregivers and their children remains low in Ghana. Nevertheless, we found that when a mother uses ITN, her children under the age of five are more likely to use it as well.

## Introduction

Globally, malaria is a public health concern, with half of the population at risk of infection [1]. Nearly one million people die each year from malaria [2]. The Plasmodium parasites cause malaria, which is transmitted when one is bitten by anopheles mosquitoes [3, 4]. Globally, malaria cases increased from 227 million in 2019 to 241 million in 2020, with most of this increase occurring in the African region [5]. According to the World Malaria Report, malaria mortality steadily declined from 896,000 in 2000 to 558,000 in 2019. However, malaria deaths increased in 2020 as compared to 2019 (558,000), reaching an estimated figure of 627,000 [5]. WHO’s World Malaria Report for 2021 also reported that 68% (47,000) of the 69,000 extra deaths were caused by problems with service delivery during the COVID-19 pandemic [5].

Due to its high morbidity and mortality rate, malaria poses a major threat to children younger than five years of age [6]. Malaria cases increased from 213 million to 228 million in the WHO African Region between 2019 and 2020, with deaths rising from 534, 000 to 602, 000. Around 95% and 96% occurred in this region, with children under the age of five accounting for 80% of all deaths [5]. In Ghana, malaria is endemic, perennial, and follows a seasonal variation, particularly in the northern part of the country. The rate of spread of malaria differs geographically, conditional on the length of the dry season (December to March), which has little transmission [7]. Ghana is among the 15 countries with the highest malaria cases, accounting for 2% of global malaria cases and 3% of malaria mortality. However, malaria cases and deaths in Ghana decreased between 2016 and 2019. The number of cases decreased by 32% from 237 in 2016 to 161 per 1000 in 2019 [8]. Malaria deaths decreased by 7%, from 0.4 deaths per 1,000 to 0.37 deaths during the same period.

Globally, 18 (58%) out of 31 countries completed the continuous distribution of insecticide-treated mosquito nets (ITNs) campaign, which resulted in 159 million (72%) ITNs being distributed in 2020 [5]. In the Ghanaian setting, both mass and continuous distribution campaigns have been employed in distributing ITN to the entire populace. The recent mass distribution campaign took place in 2018. ITNs are routinely distributed at maternity care centers, child welfare centers, and primary schools as part of the continuous distribution. ITNs are given out free to children aged 18 months or older at their second dose of the measles-rubella vaccine during child welfare visits and pregnant women during their first ANC visit as part of health facility-based distribution [9]. Ghana has 74% of households owning at least one ITN in 2019, but a low usage rate (54%) for children under five years [10]. The global malaria elimination programme classifies Ghana as being in the control phase of malaria elimination [9]. As a result, Ghana and most sub-Saharan African countries have made an effort to attain a reduced level of malaria morbidity [11]. The country’s development and implementation of consecutive strategic action plans, policy interventions, and an enhancement in financial support over the years have contributed to reducing the burden of malaria in the country [12]. However, the National Malaria Control Programme still has a lot more to achieve. It aims at achieving 100% household ownership of at least one ITN, 80% of the general population sleeping under ITNs, and increasing the number of children under five who sleep under ITNs to 85% from current levels [13].

Therefore, this study seeks to identify the determinants of ITN usage in children under five years old, specifically how the use of ITN by mothers is associated with the use by their children under five years of age, in order to improve the implementation of integrated malaria control strategies and interventions in Ghana.

### Theoretical framework

In order to encourage people to use ITN, which is a conditioned behaviour, it is necessary to comprehend their motivations. With a deeper understanding of the complex factors influencing behavioural practice, health promotion programmes and/or interventions can be successfully incorporated into people’s daily lives to bring about improvements in health behaviour [14]. The Health Belief Model (HBM) is a health-focused behavioral cognitive model [15]. ITN use is influenced by human behavioural and social aspects because ITN programmes depend on the acceptance and active participation of individuals and communities [5]. HBM comprises six dimensions, including self-efficacy, perceived benefit, perceived barrier, perceived susceptibility, and cues to action [16]. The HBM makes the assumption that if a person thinks that a bad health condition can be prevented, he or she will act in a way that is connected to their health. Similarly, the model implies that a person will act appropriately if they have a positive expectation that by taking a recommended action, they can prevent a negative health problem [17]. It has been discovered that the season, along with power dynamics within the household, influences the use of mosquito nets [18]. In an exploratory study conducted in rural Burkina Faso, Okrah et al. found that the majority of bed nets were used by adults, particularly household heads, and that they were mostly used during the rainy season [19]. According to Mugisha and Arinaitwe, the majority of children in Uganda use mosquito nets, mostly because they share a bed with their parents [20]. ITN use has been reported to lower overall child mortality by 63% in villages employing treated nets [21] and to have a 50% preventive effect against malaria outbreaks in highly endemic areas of Africa [22]. The HBM framework was utilised in this study to investigate ITN use in light of decreased malaria risk.

## Methods

In this study, the analysis is based on data collected from the 2019 Ghana Malaria Indicator Survey (GMIS). The sampling frame for the 2019 GMIS was based on the 2010 Population and Housing Census (PHC), which was done by the Ghana Statistical Service (GSS) in Ghana. Ghana created six (6) new regions in 2019, totaling 16 regions and 260 administrative districts, municipalities, and smetropolitans [23]. However, the new administrative boundaries were not available during the survey design. As a result, the sampling frame for the 2019 GMIS was based on the ten regional borders set under the 2010 PHC. The GMIS is a nationally representative cross-sectional household survey used to collect information on population-based malaria indicators for the country as a whole, for urban and rural areas separately, and for each of the ten (10) administrative regions (Western, Central, Greater Accra, Volta, Eastern, Ashanti, Brong Ahafo, Northern, Upper East, and Upper West) as defined in the Ghana 2010 Population and Housing Census (PHC). The sampling frame used for the 2019 GMIS is a frame of all census enumeration areas (EAs) created for the 2010 Population and Housing Census (PHC) and provided by the Ghana Statistical Service (GSS) [10].

The 2019 GMIS sample was stratified and selected from the sampling frame in two stages. Each region was divided into urban and rural areas; this yielded 20 strata for sampling. Samples of EAs were selected independently in each stratum in two stages. Implicit stratification with proportional allocation was achieved at each of the lower administrative levels by sorting the sampling frame within each sampling stratum before sample selection [10]. This was performed according to administrative units at different levels and by using a probability proportional to size selection in the first stage of sampling. In the first stage, 200 EAs (97 in urban areas and 103 in rural areas) were selected with a probability proportional to EA size and with independent selection in each sampling stratum. In the second stage of selection, a fixed number of 30 households were selected from each stratum to make up a total sample size of 6,000 households. The sampling details for the 2019 GMIS, data collection methods and tools, as well as the quality control measures, have been documented in the full report [10].

This study utilised the children’s file for analysis. A total of 1,876 women aged 15–49 years were included in the study.

### Dependent variable

In this study, the dependent variable was the use of ITN by children under five years of age the night before the interview. The response options were no child (coded “0”), all children (coded “1”), and some children (coded “2”). We used some children (coded “2”) as the base outcome of the dependent variable in the multinomial logistic regression. The use of ITNs the night before the interview might be tentative, as it may not reflect the true pattern of usage of ITNs by children under five. That is, respondents might not have used ITNs for some time but used them the night before the interview.

### Covariates

The following sociodemographic and economic characteristics were included in the study as covariates: respondents’ age group; highest level of education; place of residence; number of children under 5 in household; wealth index; use of ITN by the respondent; having a valid national health insurance card; the number of antenatal care (ANC) visits during pregnancy of their last child; being pregnant at the time of the interview; exposure to information on malaria within the past 6 months; and whether mothers or caregivers would allow their children under five to be vaccinated against malaria [13, 24].

The levels of wealth index were already created in the dataset. However, the wealth index was generated in the 2019 GMIS as follows:

“Scores were assigned to households based on the quantity and kind of consumer goods they own, including a television to a bicycle or automobile, as well as home factors like the source of drinking water, the availability of restrooms, and the type of flooring. With the help of principal component analysis, these scores were created. Each typical (de jure) household member is given a score, which is then used to rank each person in the household population. The distribution is then split into five equal categories, each of which has 20% of the population” [10].

### Statistical analysis

The study used descriptive and inferential statistical analyses. The descriptive statistics and Pearson Chi-square goodness-of-fit test were computed for the percentage of children under five who slept under ITN and other demographic and economic characteristics to establish the significant difference between them. We performed multinomial logistic regression to examine the determinants of ITN use in children under five years of age. We used some children (coded “2”) as the base outcome of the dependent variable in the multinomial logistic regression. We adjusted for the effect of sociodemographic and economic factors on the use of ITN by children under five. A test for multicollinearity was performed before fitting the adjusted model, and we found that there was no collinearity (mean VIF = 1.23, range = 1.01–2.04). To adjust for the complex survey design used in the GMIS, sampling weight was applied in all the analyses [25]. All statistical analyses were conducted using Stata version 15.0 (StataCorp. LP, College Station, USA). The results are presented as relative risk ratios (RRR) with their corresponding 95% confidence intervals (CIs). The statistical significance level was set at a p<0.05.

### Ethical consideration

This study analysed secondary data from the 2019 Ghana Malaria Indicator Survey (GMIS). The protocol for the 2019 GMIS was approved by the Ghana Health Service Ethics Review Committee and the Inner-City Fund’s Institutional Review Board. The authors were permitted to use the data by MEASURE DHS/ICF International. The Malaria Indicator Survey (MIS) Programme adheres to industry guidelines for protecting the privacy of respondents. ICF International guarantees that the survey complies with the Human Subjects Protection Act of the United States Department of Health and Human Services. Before the survey, the MIS project sought and received the required ethical approval. This study, therefore, did not require any additional approvals. More information on data and ethical principles can be found at http://goo.gl/ny8T6X.

## Results

### The percentage of children under five sleeping under insecticide-treated bed nets by mothers’ background characteristics

In total, 1,876 mothers/caregivers with children under five years of age participated in the study. The results showed that 28.15% of the households did not have children under five years of age sleeping under mosquito bed nets the previous night before the study; 57.94% had all children under five sleeping under bed nets the previous night; and 13.91% had some children under five sleeping under mosquito bed nets the previous night before the interview. We found significant differences in the use of ITN by children under five by all the sociodemographic, economic, and health-related characteristics except respondents’ age group (p-value = 0.275), being pregnant at the time of interview (p-value = 0.120), having a valid national health insurance card (p-value = 0.137), seeing or hearing any messages about malaria in the past 6 months (p-value = 0.289), and mothers/caregivers who would allow their children to be vaccinated against malaria (p-value = 0.198) (Table 1).

**Table 1:** The percentage of children under five sleeping under ITN by mothers’ background characteristics.

Variable	Count [%]	Percentage of children under 5 sleeping under ITN the night before the interview			P-value
		No child under five slept under ITN	All children under five slept under ITN	Some children under five slept under ITN	
All sample	1876	28.15 [24.81,31.75]	57.94 [54.14,61.65]	13.91 [11.16,17.2]	
Mother’s age group					0.153
15–24	443 [23.61]	25.04 [19.87,31.04]	63.1 [57.13,68.7]	11.86 [8.282,16.7]	
25–34	881 [46.96]	33.45 [29.17,38.03]	57.31 [52.38,62.1]	9.24 [6.875,12.3]	
35–49	552 [29.42]	30.72 [25.17,36.9]	58.18 [52.01,64.1]	11.1 [7.856,15.45]	
Mother’s level of education					**<0.001**
No education	500 [26.65]	17.92 [13.2,23.87]	61.93 [52.77,70.32]	20.15 [14.6,27.13]	
Primary	404 [21.54]	29.09 [23.13,35.87]	60.68 [54.3,66.71]	10.23 [7.10,14.52]	
Secondary	888 [47.33]	35.68 [30.97,40.69]	57.7 [52.76,62.49]	6.62 [4.79,9.07]	
Higher	84 [4.48]	44.26 [30.58,58.87]	48.5 [34.55,62.69]	7.24 [3.18,15.65]	
Place of residence					**<0.001**
Urban	681 [36.3]	46.89 [40.68,53.19]	44.03 [38.16,50.06]	9.09 [6.35,12.85]	
Rural	1195 [63.7]	20.42 [16.69,24.75]	68.4 [63.46,72.95]	11.18 [8.40,14.73]	
Wealth index					**<0.001**
Poorest	606 [32.3]	12.13 [9.47,15.4]	69.8 [63.06,75.79]	18.07 [12.88,24.76]	** **
Poorer	414 [22.07]	17.77 [13.27,23.39]	72.01 [65.05,78.06]	10.21 [6.64,15.4]	** **
Middle	383 [20.42]	33.31 [26.63,40.73]	57.67 [51.06,64.02]	9.02 [5.77,13.84]	** **
Richer	266 [14.18]	45.8 [37.6,54.23]	46.23 [38.02,54.64]	7.98 [3.81,15.93]	** **
Richest	207 [11.03]	56.91 [46.6,66.66]	39.2 [29.39,49.96]	3.89 [1.91,7.76]	** **
Number of children under 5 in household					**<0.001**
1	941 [50.16]	37.87 [33.26,42.71]	61.53 [56.64,66.19]	0.61 [.238,1.54]	
2	625 [33.32]	27.85 [23.79,32.3]	62.9 [58.49,67.1]	9.26 [6.97,12.19]	
3+	310 [16.52]	13.97 [9.087,20.86]	41.6 [32.69,51.1]	44.43 [35.59,53.64]	
Being pregnant at the time of interview					0.327
No	1714 [91.36]	30.31 [26.56,34.34]	59.07 [55.01,63]	10.63 [8.47,13.26]	
Yes	162 [8.64]	36.09 [27.01,46.28]	56.37 [46.02,66.19]	7.55 [4.20,13.19]	
Mother/caregiver slept under a mosquito net					**<0.001**
No	553 [29.48]	79.85 [74.98,83.98]	14.32 [10.99,18.45]	5.83 [3.91,8.60]	** **
Yes	1323 [70.52]	5.27 [3.72,7.4]	82.02 [78.38,85.16]	12.72 [10.13,15.85]	** **
Has a valid national health insurance card					0.086
No	683 [39.99]	34.31 [28.79,40.29]	58.19 [52.46,63.71]	7.50 [5.20,10.71]	
Yes	1025 [60.01]	29.27 [25.61,33.23]	59.36 [54.97,63.6]	11.37 [8.39,15.22]	
Number of ANC visits during most recent pregnancy					**<0.001**
None	41 [2.19]	13.59 [6.347,26.75]	64.65 [47.63,78.62]	21.76 [10.95,38.61]	
1 to 3	129 [6.88]	28.95 [18.57,42.13]	54.52 [42.87,65.7]	16.53 [10.61,24.84]	
4 to 7	957 [51.01]	26.72 [22.9,30.93]	60.9 [56.34,65.28]	12.38 [9.411,16.12]	
8+	749 [39.93]	36.76 [31.51,42.33]	56.93 [51.48,62.21]	6.32 [4.08,9.66]	
Seen/heard of malaria messages in the past 6 months					0.513
No	833 [44.4]	30.01 [25.33,35.16]	60.67 [55.34,65.76]	9.32 [6.99,12.32]	
Yes	1043 [55.6]	31.37 [27.13,35.95]	57.55 [52.9,62.06]	11.08 [8.30,14.65]	
Willingness to allow child(ren) to be vaccinated against malaria					0.307
No	94 [5.01]	30.83 [20.07,44.17]	52.1 [37.44,66.4]	17.07 [7.829,33.29]	
Yes	1782 [94.99]	30.81 [27.21,34.67]	59.17 [55.34,62.9]	10.01 [7.90,12.61]	

### Association between children under five sleeping under ITN and background characteristics of their mothers or caregivers

The use of bed nets in children under five was categorised as “no child under 5 slept under ITN the previous night”, “all children under 5 slept under ITN the previous night” and “some children under 5 slept under ITN the previous night (the base outcome).” The results indicate that women who slept under mosquito bed nets were more likely to have their children under five sleeping under mosquito bed nets. In the multinomial logistic regression model, we adjusted for the effect of sociodemographic, and economic factors on children under five sleeping under mosquito bed nets the night before the study. The results showed that mothers/caregivers who slept under a mosquito bed net were less likely to have no child sleeping under a mosquito bed net (RRR = 0.031; 95% CI: 0.015, 0.059). However, they were more likely to have all children sleeping under a mosquito bed net (RRR = 2.468; 95% CI: 1.477, 4.123) compared to having some children sleeping under a mosquito bed net last night.

Again, we found that the mothers/caregivers who made 4 to 7 ANC visits during pregnancy were 19% less likely to have a child sleep under ITN as compared to those who made no ANC visits. Wealthier mothers and caregivers are more likely to allow no child to sleep under ITN.

Moreover, mothers/caregivers with at least two (2) children under the age of 5 were less likely to have no child and all children sleep under ITN. Whereas respondents who are willing to allow their children under five to be vaccinated against malaria were 3.22 times more likely to have no child sleep under ITN last night. On the other hand, the mother/caregiver’s age, education, place of residence, having a valid national health insurance card, being pregnant at the time of the interview, and having seen or heard of any messages about malaria in the past 6 months were not significantly associated with children under 5 sleeping under mosquito bed nets (Table 2).

**Table 2:** Association between children under five sleeping under ITN and background characteristics of their mothers or caregivers (Base outcome (reference group) = Some children slept under ITN the previous night).

Variables	No child under 5 slept under ITN the previous night		All children 5 slept under ITN the previous night	
	RRR [95% CI]	P-value	RRR [95% CI]	P-value
Respondents’ Age group				
15–24	1			
25–34	1.82 [0.90, 3.66]	0.093	1.19 [0.66, 2.15]	0.552
35–49	1.51 [0.68, 3.34]	0.309	0 .99 [0.56, 1.74]	0.958
Respondents’ Education				
No education	1			
Primary	1.62 [0.76, 3.49]	0.213	1.52 [0.78, 2.97]	0.218
Secondary	1.66 [0.70, 3.90]	0.247	1.62 [0.90, 2.92]	0.106
Higher	0.44 [0.11, 1.84]	0.261	0.95 [0.23, 3.91]	0.937
Number of children under 5 in household				
1	1			
2	0.08 [0.03, 0.22]	**<0.001**	0.08 [0.03, 0.22]	**<0.001**
3+	0.01 [0.01, 0.03]	**<0.001**	0.01 [0.00, 0.03]	**<0.001**
Place of residence				
Urban	1			
Rural	1.46 [0.67, 3.17]	0.339	1.66 [0.86, 3.20]	0.165
Wealth index				
Poorest	1			
Poorer	2.60 [1.02, 6.67]	**0.047**	1.97 [0.97, 4.04]	0.062
Middle	2.91 [0.87, 9.73]	0.083	1.09 [0.46, 2.58]	0.844
Richer	4.00 [1.26, 12.68]	**0.019**	1.40 [0.56, 3.51]	0.468
Richest	6.43 [1.56, 26.47]	**0.01**	1.52 [0.42, 5.48]	0.516
Mother/caregiver slept under a mosquito net				
No	1			
Yes	0.03 [0.02, 0.06]	**<0.001**	2.47 [1.48, 4.12]	**0.001**
Has a valid national health insurance card				
No	1			
Yes	0.65 [0.32, 1.32]	0.229	0.85 [0.51, 1.41]	0.526
Number of ANC visits during most recent pregnancy				
None	1			
1 to 3	0.28 [0.04, 1.83]	0.184	0.35 [0.07, 1.78]	0.201
4 to 7	**0.19 [0.04, 0.93]**	**0.041**	0.29 [0.06, 1.29]	0.106
8+	0.39 [0.08, 1.92]	0.243	0.48 [0.11, 2.14]	0.332
Being pregnant at the time of the interview				
No	1			
Yes	1.15 [0.52, 2.53]	0.732	1.14 [0.59, 2.24]	0.692
Seen or heard any messages about malaria in the past 6 months				
No	1			
Yes	0.68 [0.37, 1.26]	0.216	0.79 [0.47, 1.32]	0.367
Willingness to allow child(ren) to be vaccinated against malaria				
No	1			
Yes	3.22 [1.12, 9.30]	**0.031**	2.24 [0.82, 6.10]	0.116

## Discussion

This study assessed the association between the use of ITN in children under five and their mothers/caregivers using nationally representative data from the 2019 MIS in Ghana. The study found that 58.59% and 61.88% of mothers/caregivers and children under five years old, respectively, slept under a mosquito bed net the night before the interview. This result is similar to a study conducted in Burkina Faso, which found that two-thirds (67.0%) of children under five sleep under the ITN [26]. In contrast, a study conducted in Nigeria that compared the level of usage among mothers/caregivers and children under five indicated that 49.1% of women and 43.1% of children under five slept under ITN the previous night before the conduct of the study [27] and 45.3% of women and 37.5% of children under five slept under ITN the previous night before the conduct of the study [28]. These are substantiated by the fact that, in Ghana and the majority of African nations, the Ministry of Health has made concerted efforts to provide education on malaria prevention with a strong emphasis on the use of ITN as the simplest and most effective method, with the assistance of WHO and UNICEF [29].

In the current study, it was found that the use of ITN in children under five years of age improved over the 2008 and 2014 Ghana Demographic and Health Surveys, which reported 41% and 47%, respectively [30, 31]. This improvement may be attributed to the effort of the malaria control programme in the country towards the universal coverage target of 80% and above [32]. Other studies also reported increases in the usage of ITN [33, 34]. However, about 40.0% of children did not use ITN on the night before the conduct of the interview in the country. This may be due to the high room temperature [35–37]; the use of other malaria preventive methods such as repellants [38]; and the poor quality of ITN [39].

After controlling for a number of factors, our findings showed that mothers/caregivers who slept under a mosquito bed net would also have their children under five sleep under a mosquito bed net. Thus, mothers/caregivers who slept under ITN were less likely to have no child sleep under ITN (RRR = 0.019; 95% CI: 0.009, 0.039). This implies that, in as much as mothers/caregivers sleep under ITN, it is less likely that any child under the age of five of theirs would not sleep under ITN. Similarly, mothers/caregivers who slept under ITN were more likely to have all their children sleeping under ITN (RRR = 2.084; 95% CI: 1.074, 4.044) compared to having some children sleeping under ITN. A previous study reported that children under five whose mothers use ITN are more likely to use them too [20]. Malaria knowledge was associated with the use of ITN among mothers of children under the age of five in Ethiopia [40].

Households with at least two children under five years of age were less likely to have no child and all children sleeping under ITN as compared to some children sleeping under ITN. Our study implies that households with at least two children under five would have at least some children sleeping under ITN. That is, at least there are some households with an unequal distribution of ITN with regard to household size, which significantly lowers the use of ITN [41]. This finding is similar to a study conducted in the Gambia and Ghana [31, 32].

Furthermore, compared to some children, mothers and caregivers who attended ANC visits 4–7 times during pregnancy were less likely to have had no child sleep under ITN. This implies that mothers or caregivers who attended ANC visits four to seven times would have at least one child under the age of five who used ITN. Similar findings have been reported among Senegalese [42]. ITNs are given to pregnant women at no cost during ANC visits as a vital approach for controlling malaria and increasing coverage and use by both pregnant women and their children under five [9, 43–45]. In most areas where malaria is endemic, women sleep with their infants through the first year of life as a protective effect of ITNs delivered to pregnant women during ANC [33, 35].

Moreover, socio-economic status is a key variable in assessing the effect of health interventions or programmes in society. It is an indication as to whether the interventions reach the poor or vulnerable groups relative to the well-to-do people in societies. We found that wealthier mothers/caregivers had a higher chance of letting none of their children under five sleep under ITN compared to some children sleeping under ITN. The finding is also consistent with reports from Ghana [32] and Senegal [42]. Also, the Ghana Statistical Service indicated that the percentage of women who sleep under ITN tends to decrease with wealth [31]. However, Asumah et al. found that increasing average monthly income was associated with the utilisation of ITNs in the Kasena-Nankana East Municipality in the Upper East of Ghana [29]. The distinctiveness of each setting and the ITN deployment interventions and strategies may result in a disproportionate order between the well-off and relatively poor categories. As a result, wealthier households are more likely to have access to other malaria prevention strategies and may not use ITN even if they have them [32, 46]. Mothers/caregivers who would allow their children to be vaccinated against malaria were 3.22 times more likely to have no child sleeping under an TN as compared to some children using an aITN (RRR = 3.22; 95% CI = 1.117, 9.304). That is mothers/caregivers would feel their children under five would be protected from malaria after the vaccination.

### Implications of the current study’s findings for malaria control policy and practise in Ghana

The current study’s findings have implications for malaria control policy and practise in Ghana. Despite increased household ownership of ITN in Ghana, usage by vulnerable groups such as children under the age of five remains low, as evidenced by the current study and other national reports [10]. The current study, on the other hand, identified some key factors that can be addressed to encourage the use of ITN. The use of ITN by children under the age of five is influenced in part by its use among mothers or caregivers. As a result, increased advocacy for the use of ITN by mothers or caregivers of children under the age of five is required to improve usage among children. ANC provides an avenue for health education and interactions with families to promote positive health behaviours.

### Strengths and limitations

We used a nationally representative sample and robust statistical techniques to generate estimates that are generalizable to the entire Ghanaian population of children under five years of age. These findings are important for decision-makers and stakeholders. However, we acknowledge that some limitations also exist. The GMIS is a cross-sectional survey, and the conclusions are limited to statistical associations rather than causal relationships. In addition, the questionnaire relied on self-reported responses, which are prone to recall bias and socially desirable answers. Furthermore, the use of ITN was based on the previous night before the conduct of the study, which might be tentative, as it may not reflect the true nature of the usage of ITN in children under five. That is, respondents might not be using ITN but used it the night before the study was conducted.

## Conclusion

The study identified that about three-fifths of both women and children under five slept under ITN. Again, it was found that women who slept under ITN were more likely to have their children under five sleeping under ITN too. ITNs are given to pregnant mothers/caregivers freely during ANC visits as a vital approach for controlling malaria and increasing coverage and use by their children under five. Thus, the target of achieving 85% utilisation of ITN in children under five under the National Malaria Control Programme in Ghana should include usage by their mothers.

## Abbreviations

ANC: Antenatal Clinic
DHS: Demographic and Health Survey
EAs: Enumeration Areas
GHS: Ghana Health Service
GMIS: Ghana Malaria Indicator Survey
GSS: Ghana Statistical Service
HBM: Health Belief Model
ITN: Insecticide Treated Net
MIS: Malaria Indicator Survey
PHC: Population and Housing Census
RRR: Relative Risk Ratio
